# A review of cell-free DNA and epigenetics for non-invasive diagnosis in solid organ transplantation

**DOI:** 10.3389/frtra.2024.1474920

**Published:** 2024-11-15

**Authors:** Alizée Sebastian, Monique Silvy, Benjamin Coiffard, Martine Reynaud-Gaubert, Frédérique Magdinier, Jacques Chiaroni, Christophe Picard, Pascal Pedini

**Affiliations:** ^1^ Aix-Marseille University, Centre National de la Recherche Scientifique (CNRS), Etablissement Français du Sang (EFS), Anthropologie Bio-Culturelle, Droit, Éthique et Santé (ADES), Marseille, France; ^2^ BeDia Genomics, Aix-en-Provence, France; ^3^ Department of Respiratory Medicine and Lung Transplantation, Aix-Marseille University, APHM, Hôpital Nord, Marseille, France; ^4^ Aix-Marseille University, Institut National de la Santé Et de la Recherche Médicale (INSERM), Marseille Medical Genetics (MMG), Marseille Medical Genetics, Marseille, France; ^5^ Immunogenetics Laboratory, Etablissement Français du Sang, Marseille, France

**Keywords:** cfDNA, epigenetic, transplantation, biomarker, diagnosis, methylation

## Abstract

**Introduction:**

Circulating cell-free DNA (cfDNA) is emerging as a non-invasive biomarker in solid organ transplantation (SOT) monitoring and data on its diagnostic potential have been increasing in recent years. This review aims to summarize the main advances in technologies, clinical applications and future perspectives of cfDNA for transplantation, and to approach the contribution of epigenetics to improve the specific detection of rejection.

**Methods:**

Published literature investigating cfDNA as a biomarker for the diagnosis of transplant rejection was systematically reviewed, specifically clinical trials evaluating the test performance of algorithms predicting rejection based on cfDNA fraction. Literature highlighting epigenetic features in transplant rejection was also reviewed to outline the potential contribution of the epigenomic analysis to the needs of rejection-specific diagnosis.

**Results:**

40 articles were reviewed, and results were extracted and summarized. 16 met the inclusion criteria by evaluating the diagnostic performance of a predictive test for the discrimination of rejection vs. non-rejection patients (2 heart, 3 liver, 4 kidney, and 7 lung transplantations). The recurring conclusion is the kinetics of dd-cfDNA levels, strongly increasing immediately after transplantation and reaching basal levels after days to weeks and remaining stable in non-rejection patients. On the other hand, rejection is characterized by an increase in dd-cfDNA levels, depending on the transplanted organs. In addition, the epigenetic signature can help improve the specificity of the diagnosis of rejection by searching for specific epigenetic features that are by the clinical status of patients.

**Conclusion:**

Cell-free DNA is a promising non-invasive biomarker but still needs standardization of technologies and protocols to be used for diagnostic purposes. Moreover, the lack of specificity of this marker can be compensated by the contribution of epigenetic analysis for which data are growing, although progress is still needed for its use in a clinical context.

## 1 Introduction

Circulating cell-free DNA (cfDNA) is emerging as a non-invasive biomarker in the monitoring of solid organ transplantation (SOT). Unlike traditional follow up methods, cfDNA offers a safer and cheaper option to detect acute or chronic rejection. However, analytical and technical challenges remain, particularly for the sensitivity and specificity of cfDNA detection. Epigenetic approaches based on the analysis of the regulation of gene expression is a significative contribution. Epigenetic signature specific to the transplant allows to differentiate precisely the different cfDNA sources increasing specificity and early detection of rejection. Combining epigenetic analysis to cfDNA detection in the plasma of patients could improve patients' post-transplantation monitoring and help to avoid complications and late diagnosis.

### 1.1 Cell-free DNA

The discovery of cell-free DNA (cfDNA) in the serum of cancer patients in 1948 (1) represented a major advance in biology and opened possibilities of applications in medicine. For many years, however, the lack of sensitive analytical techniques delayed further study of cfDNA. In 1965, cfDNA was proposed as a potentially relevant biomarker in oncogenesis (2) and, over time, thanks to the development of liquid biopsies, other areas of medicine became interested in cfDNA, such as autoimmune diseases, organ transplantation or fetal medicine.

In recent years, the biology of cfDNA has been studied from two perspectives: quantification, whether absolute or relative, and qualification such as cellular origin (nuclear or mitochondrial DNA), tissue origin, production mechanism, fragment size, epigenetic markers, and so on. In healthy individuals, cfDNA originates from apoptosis during cell renewal (1–3), and from active cellular secretion (4, 5).

Apoptosis was thought to be the main cause of cfDNA release considering its capital role in cell homeostasis and renewal (6, 7) justified by the non-random fragmentation pattern of cfDNA (1), mirroring apoptotic patterns. Indeed, in 1984, the ladder-like electrophoretic pattern of apoptotic DNA is found in cfDNA (8) suggesting an intervention of apoptotic enzymes in the cfDNA release processes (1, 6). cfDNA fragments range from 80 to 200 bp (5), with most fragments around 166 bp (2, 9, 10) corresponding to the length of the DNA wrapped around histones. Other release mechanisms have been identified thus far but several remain misunderstood especially for the fragment size of DNA produced or the contribution to the cfDNA pool of each of them. Necrosis would contribute to large cfDNA fragments (1,000 to 10,000 bp) because of random DNA fragmentation (11), but also smaller fragments resulting from nuclease cleavage of these long fragments. Other suggested release processes include erythroblast enucleation and NETosis, but these remain poorly understood (6).

At present, the best-known characteristic of cfDNA is its quantification in plasma. Most studies agree that changes in cfDNA levels would be representative of a biological change in individuals, which can be significant enough to diagnose an abnormal state. Physiological cfDNA levels vary between 0 and 100 ng/ml inter and intra individually, over time, and are influenced by physical exercise, inflammation, or tissue injury in healthy individuals (12, 13). It is cleared from blood in 16 min to 2.5 h through three mechanisms: DNases cleavage in the blood, renal filtration, and mainly liver elimination (1, 3, 11). The dynamic nature and shortness of its lifespan in blood raises the first challenge, as the increased levels of cfDNA following an event may already have returned to baseline when sampling. On the other hand, this characteristic can be taken as a positive point, if considering that the occurrence of an event leads to a rapid change in cfDNA levels and allows, for example, to adapt a treatment as quickly as possible.

### 1.2 Clinical applications of cfDNA

When studies started to show differences in plasma cfDNA in pathological contexts, new potential diagnosis approaches began to appear. In 1966, quantifications of cfDNA in lupus erythematosus and rheumatoid arthritis patients compared to healthy individuals showed variations in cfDNA quantity (14, 15). Later in 1977 in the field of oncology, Leon et al. quantified cfDNA through radioimmunoassay in several types of cancer, highlighting a cfDNA increase in cancer patients. Higher cfDNA levels are observed in metastatic cases, decreasing after radiation therapy except in treatment non-responding patients (16). Genetic alterations from tumor were identified in plasma cfDNA, suggesting that DNA released by cancerous cells can be relevant in cancer diagnosis and monitoring (17, 18). No diagnostic test received approval by health instances, but recently the European Society for Medical Oncology (ESMO) published guidelines for the use of ctDNA for genotyping advanced cancers and help therapy decision-making for patients as an alternative strategy to tumor-based approaches (19). The United States Food and Drug Administration also published in 2022 guidelines intended to industries for the use of ctDNA as a biomarker of cancer in clinical trials for the development of drugs in early-stage solid tumor malignancies (20).

Another key application of cfDNA concerns non-invasive prenatal diagnostic (NIPD), using cell-free fetal DNA (cffDNA) in the plasma and urine of pregnant women. Lo et al. showed in 1998 that maternal plasma contains high concentrations of cffDNA detectable as early as at the 7th week of gestation (21). Since then, cffDNA is used to directly analyze the genomic information of the fetus and determine fetal sex, assess the RhD, aneuploidies, microdeletions or detect paternally inherited genetic disorders (22, 23). The use of cfDNA improved the safety of prenatal testing by avoiding invasive obstetric procedures with risks of miscarriage (22).

Finally, the clinical application of cfDNA that will be discussed in this review, relates to organ transplantation. Currently, the monitoring of solid organ transplantation (SOT) is performed by biopsy, clinically indicated in suspicion of rejection or part of the follow-up of the transplant in the absence of symptoms (24). However, biopsies present limitations, and from this perspective, non-invasive diagnosis tools such as cfDNA would be highly relevant. To explore the opportunities and limitations of cfDNA diagnosis in SOT, 40 articles were reviewed on the topic of organ transplantation, 16 of which met the inclusion criteria by evaluating the diagnostic performance of a predictive test for the discrimination of rejection vs. non-rejection patients.

### 1.3 cfDNA exploration techniques

The workflow for handling cfDNA samples has greatly evolved resulting in the increase of the quantity and quality of the cfDNA collected. Yet, pre-analytical treatment from blood sample to purified cfDNA is still not standardized (25). Nonetheless, steps remain similar between studies, and it is possible to summarize protocols as follows.

First, the blood must be collected in specific tubes to preserve nucleated cells and prevent cell lysis and blood coagulation. Plasma separation has to be performed as soon as possible by a double centrifugation at 1,600 × g then 16,000 × g during 10 min each and will be conserved at −80 °C until cfDNA extraction (25). cfDNA yields are shown to vary from 4 weeks of storage and it is thus advised to perform analysis on cfDNA prior (26, 27). Different protocols exist for cfDNA extraction from plasma, based on magnetic beads or columns and is the most impactful parameter in pre-analytical process of cfDNA. A comparative study between four (semi) automated extraction showed significative differences in cfDNA yields depending on the extraction used (28).

For quantification, the low amount of cfDNA in plasma requires sensitive techniques for its analysis. PCR-based methods have been developed such as quantitative real-time PCR (qPCR), and more recently digital-PCR (dPCR). The contribution of dPCR is the partition of the PCR reaction to thousands of droplets (droplet-digital PCR) or chambers to increase the probability of detection of rare events. Digital PCR allows great sensitivity and can then be used with low DNA input, suitable for cfDNA.

## 2 cfDNA quantification in transplantation

Biopsy is considered as the “gold standard” for follow-up in all types of solid organ transplantation (SOT). However, it is an invasive and risky procedure for patients, in addition to being costly and unreliable due to the inter-observer variability. It exposes patients to potential infections and complications (complication rate of about 1% in kidney, heart, and liver transplantations), not to mention the fact that this procedure often fails to detect rejection early enough. Approximately 25% of biopsies result in an insufficient sample to diagnose the onset of rejection (24, 29–31). These limitations encouraged the development of alternative methods like the assessment of gene expression profiling in heart transplantation, the liver enzymes dosage in liver transplant recipients or the serum creatinine dosage in kidney recipients. However, none of these alternative methods can specifically detect rejection and are only a mirror of the organ functional state (24, 29, 30, 32).

### 2.1 Quantification of dd-cfDNA

In 1998, it was suggested that DNA from the transplanted organ was present in the plasma of recipients and it was shown that donor-specific sequences can be found in the plasma of liver and kidney transplant recipients (21). This was the first description of donor-derived cell-free DNA (dd-cfDNA) whose increase in the plasma and urine of SOT recipients is proven to be a result of cell damage in the transplant and can therefore be used as a biomarker of graft health and integrity (33) through quantification and qualification of dd-cfDNA.

Several methods are used for relative and absolute quantification of dd-cfDNA, most of which are PCR-based, including qPCR and dPCR. Historically, the first relevant method to identify dd-cfDNA in recipient's plasma was the detection of Y-specific genetic sequences in the plasma of female recipients of male organ donor. However, this required a gender-mismatch and is therefore only applicable to a specific group of the transplanted population (33). Human Leukocyte Antigens (HLA) mismatches have also been exploited but this requires genotyping of both donor and recipient, which is sometimes difficult to obtain. A more universal approach is the analysis of single nucleotide polymorphisms (SNPs) using high throughput sequencing to detect informative SNPs and assess the minor type of DNA using computational approaches, which allows to dispense from genotyping (24, 34). PCR techniques are commonly used to detect and quantify cfDNA and dd-cfDNA considering its sensitivity. Today, dPCR is increasingly used to study cfDNA as it is designed to detect rare events with a great sensitivity. Moreover, the determination of dd-cfDNA by qPCR or dPCR techniques is specific because selected markers from the donor and recipient are previously tested on a pre-transplant sample. The speed with which the results can be obtained is an advantage, making it possible to deal with emergencies in transplant medicine. On the other hand, NGS is more accurate than dPCR, but unsuitable for emergency and require enough cfDNA. The absolute quantification of dd-cfDNA can be expressed as copy number or genome equivalent per milliliter (cp/ml or GE/ml respectively), while relative quantification, calculated as the percentage of dd-cfDNA in the total cfDNA pool is the most used.

### 2.2 Ischemia-reperfusion injury

The first important event in transplantation is organ reperfusion after hours of ischemia, which inevitably induces ischemia-reperfusion injury (IRI) and damages the transplanted cells (35), resulting in the release of cfDNA. This dd-cfDNA peak is reported in all types of SOT, and does not persist for long before decreasing to basal levels, which depends on the organ type (9, 32, 36, 37) (Table 1).

**Table 1:** Recapitulative table of dd-cfDNA levels in the diverse types of solid organ transplantations.

Organ	Relative dd-cfDNA peak levels after surgery	Relative basal dd-cfDNA levels	Time to reach basal levels	Sources
Kidney	10–20%	<1%	5–10 days	Shen et al. (38) Beck et al. (39) Gielis et al. (40)
Liver	Up to 90%	<10%	10 days	Schütz et al. (36) Zhao et al. (32)
Heart	2.8%	<0.5%	5 days	Beck et al. (39) Agbor-Enoh et al. (41)
Lung	26%	<2%	1–4 months	De Vlaminck et al. (42) Jang et al. (43) Sorbini et al. (37)

Liver is the most vulnerable organ to IRI. The extension of selection criteria because of shortage and the growing waiting list results in higher risk of graft-associated complications (44, 45). cfDNA levels reach 90% after reperfusion, with basal levels <10%–15% reached after 10 days (32, 36). Kidney IRI induces an increase to 10%–20% of dd-cfDNA the day following transplantation, decreasing to <1% after 5–10 days (31, 38, 39). Of note, non-stable patients seem to maintain elevated dd-cfDNA as a hallmark of allograft injury, as patients that are not stable 10 days after transplantation already showed persistent high dd-cfDNA from the first day following transplantation (40). Early high levels of dd-cfDNA could be a clue of future complication in kidney transplantation. The heart is the organ that releases the least amount of cfDNA with concentration peaks reaching less than 3% (41) decreasing in a logarithmic way, with basal levels <0.5% in 5 days (39), down to 0.01% after 2 months (41). Lung is the organ where dd-cfDNA decreases the slowest over time, with basal levels reached after 1.5–4 months after a high increase of dd-cfDNA levels up to 26% the days following transplantation (37, 42, 43).

Interestingly, some studies reported a slight increase of dd-cfDNA months to years after transplantation, in heart (41) and lung (42, 43) transplantations. In the latter, the slight increase of dd-cfDNA after a few months have been linked to the settling of chronic injury caused by the loss of pulmonary function (43).

### 2.3 Acute rejection diagnosis

After IRI, the second event that can damage the transplant is acute rejection (AR). AR, including acute cellular (ACR) and antibody-mediated rejection (AMR), has been proven to be correlated with a significant elevation of dd-cfDNA levels in heart, liver, kidney, and lung transplantation. As a purposely harmful reaction initiated by the immune system towards the transplant, AR causes cell death and therefore cfDNA release into the bloodstream. Levels of dd-cfDNA are therefore a reflection of the organ state of acceptance by the immune system. Studies were conducted to statistically determine a cutoff value of dd-cfDNA, allowing an early discrimination of stable patients from patients developing acute rejection. These predictive models are evaluated with parameters like sensitivity, specificity, and sometimes positive and negative predictive value (PPV, NPV) to assess their ability to correctly classify patients based on observations. The key parameters of the studies are reported in Table 2.

**Table 2:** Summary table of %dd-cfDNA cutoff values suggested in studied publications.

Organ	Source	ddcfDNA AR patients	ddcfDNA stable patients	ddcfDNA cutoff value	Specificity	Sensitivity	PPV	NPV
Heart	Agbor-Enoh et al. (41)	0.38%	0.03%	–	–	–	–	–
	Kim et al. (46)	0.58%	0.04%	0.15%	76.9%	78.5%	25.1%	97.3%
Liver	Schütz et al. (36)	29.6%	–	10%	92.9%	90.3%	–	–
	Baumann et al. (47)	25%(1)	3.40%	10%	90.0%	86.0%	–	–
Liver (pediatric)	Zhao et al. (32)	41.7%	11.20%	–	–	–	–	–
Kidney	Bloom et al. (29)	AMR 2.9% ACR 1.2%	0.30%	1%	–	–	–	–
	Oellerich et al. (31)	0.57%	0.29%	–	–	–	–	–
	Bunnapradist et al. (48)	–	–	1% + 78cp/ml (2)	87.5%	100%	–	–
Kidney-pancreas	Vantura-Aguiar et al. (49)	0.83%	0.30%	–	93.0%	85.0%	85.7%	93.7%
	De Vlaminck et al. (42)	15%	–	1%	73.0%	100%	–	–
	Jang et al. (43)	0.4–0.7%	0.21%	0.5%	65.0%	95.0%	51.0%	96.0%
				1%	84.0%	77.0%	64.0%	90.0%
	Keller et al. (3)	–	–	0.54% (single-lung) 1.1% (double-lung)	–	–	–	–
	Sorbini et al. (37)	7.8%	2.2%	1.25%	73.3%	80.7%	–	–
	Ju et al. (50)	2.17%	0.7%	1.17%	86.0%	89.0%	64.0%	96.0%
	Sayah et al. (51)	1.52% (3)	0.49%	0.87%	52.0%	73.0%	34.0%	85.0%
	Pedini et al. (9)	–	–	1.72% (4)	–	–	75.0%	91.4%

(1) T-cell mediated rejection patients; (2) Authors combined two cutoff values of ddcfDNA to improve test performance; (3) Acute cellular-rejection patients; (4) Cutoff value established to discriminate between injured vs. non-injured patients, injury being either infection, rejection, or both.

Thus, the dd-cfDNA percentages are around 4-fold higher in AR liver recipients compared to stable patients. Median dd-cfDNA reaches around 30%–40% in AR patients, vs. 11% in non-rejection patients. A cutoff value of 10% dd-cfDNA is suggested to identify liver transplant rejection against stability, yielding good specificity and sensitivity values (>90% and >86%, respectively) (32, 36, 47).

In 2017, dd-cfDNA in kidney recipients has been shown to discriminate between AMR, ACR, and non-rejection patients (29). The donor cfDNA fraction is around 0.6%–0.8% for both rejection groups, 2-fold higher compared to non-rejection patients for which authors measured 0.3% (31, 49). Moreover, non-stable patients 10 days following transplantation showed higher dd-cfDNA levels initially and during the first three months (40). Bunnapradist et al. suggested the use of two cutoff values to discriminate rejection, relying on relative and absolute values of dd-cfDNA. Authors compared the predictive test performances when using a unique threshold vs. the combination of two. Using cutoff values of 1% and 78 cp/ml, sensitivity is increased compared to the use of only the relative value (100% vs. 77.8% respectively), while specificity decreases (90.6% vs. 87.5% respectively) (48). Of note, it is important to be careful with sensitivity and specificity, the aim being to have a good balance between both, while keeping good test performances. Sensitivity is crucial to diagnose rejection using dd-cfDNA and should not necessarily be set aside for the benefit of specificity.

In heart transplantation, median dd-cfDNA levels are reported 13- to 15-fold higher in AR diagnosed patients compared to stable patients (41, 46). With 0.15% of dd-cfDNA used as a threshold value to stratify rejection vs. non-rejection patients, the test performed with a Positive Predictive Value (PPV) of 25%, 97% for Negative Predictive Value (NPV), 78% sensitivity and 77% specificity (46). Moreover, cardiac allograft vasculopathy (CAV) has been reported to correlate with elevated dd-cfDNA levels. Among two groups of patients considered low (<0.12%) and high (≥0.12%) dd-cfDNA, 63% of high dd-cfDNA patients developed CAV, vs. 35% in the low dd-cfDNA group. Another interesting point is 25% of high dd-cfDNA group patients had *de novo* donor-specific antibodies (DSA) vs. 3.8% in the low dd-cfDNA group (52). This study suggests that dd-cfDNA may also be linked to DSA, and other transplant survival-threatening conditions in addition to AR.

In lung transplantation, De Vlaminck et al. Reported a significant increase in dd-cfDNA in AR and chronic lung allograft dysfunction (CLAD). Moreover, CMV (cytomegalovirus) infection, which is the greatest infectious threat after lung transplantation, causes a significant increase in dd-cfDNA, which is not found in other infections (42, 43). AMR is associated with a more important allograft injury, assessed with spirometry and dd-cfDNA increase (5.4%), able to detect the onset of rejection about 3 months before the clinical diagnosis (43, 53), and correlated to the concomitant rise of DSA levels (53). Even though the detection of DSA in AMR showed higher dd-cfDNA, DSA detection alone is not shown to be associated with an increase in dd-cfDNA (43). Most studies in lung transplant recipients report a great sensitivity to detect AR, reaching 80%–100% with thresholds of dd-cfDNA around 0.87%–1.25% (37, 42, 50, 54). In addition to detect AR vs. non-AR patients, dd-cfDNA levels correlate with the lung allograft dysfunction, assessed with FEV1 (forced expiration volume in 1s) (42, 43) and spirometry (53).

Interestingly, several studies reported the ability to detect the onset of rejection weeks before the first clinical manifestations in liver (36, 55) and lung (43, 53) transplantations, further supporting the relevance of dd-cfDNA for diagnosis.

### 2.4 Experimental limits

Some limitations must be considered in the development of dd-cfDNA as a biomarker for the diagnosis of organ rejection.

Physiological factors can impact dd-cfDNA levels and bias the diagnosis. As an example, in renal transplantation, dd-cfDNA levels are significantly different depending on vital status of the donor, linked to the differences in ischemia-reperfusion of kidneys. Thus, early dd-cfDNA percentages in recipients of deceased donors are up to 4-fold higher than in recipients of living donor after the organ's perfusion, and up to 2-fold higher in the long term (31, 32, 38).

Moreover, the biopsy procedure can cause iatrogenic injuries leading to the release of dd-cfDNA, as shown in a study of 113 paired before/after biopsy plasma samples in heart transplant recipients. Authors noted a 1.3-fold increase in dd-cfDNA when plasma was collected after biopsy (56).

In lung transplantation, another important parameter to consider is whether the transplantation is single or bilateral. Since dd-cfDNA levels were shown to differ significantly between both (9, 54). This difference could be linked to the organ's mass (24). Additionally, De Vlaminck et al. reported a cell turn-over rate of 107 cells/s in bilateral lung transplant, vs. 58 cells/s in single lung transplantation (42). It is therefore important to be careful, in clinical trials particularly, not to compare patients who have received a single or double transplant with each other, and not to use the same ddcfDNA cutoff values.

Experimental bias can also interfere with the ability to use dd-cfDNA as a biomarker of rejection. dd-cfDNA levels vary with the extraction yields of each method (28), generating experimental differences according to the methods used. Results from different studies can therefore be difficult to compare. Moreover, a crucial point of these studies is that they rely on the biopsy confirmation of the rejection state of the organ. However, the poor reliability of biopsies can lead to false negative and distort the reported test performances. Finally, dd-cfDNA levels being a ratio, they can be impacted by the recipient's cfDNA levels, that may increase in various circumstances of everyday life, and therefore minimize or maximize the reality of potential allograft injuries.

Some suggest analyzing the change value of dd-cfDNA would be more representative of the organ's dynamics over a certain time course. Agbor-Enoh suggested in 2019 a stratification of patients according to the decay kinetics of dd-cfDNA during the first 3 months following transplantation. The classification was consistent with the probability of rejection episodes, organ failure, development of donor-specific antigens (DSA), chronic lung allograft dysfunction (CLAD), or death during the first three months (54). This stratification may help to identify high-risks patients while considering the dynamics of the allograft injury. The reference change value (RCV) of dd-cfDNA is suggested to be a better biological indicator of abnormalities (57). To test this hypothesis, patients were stratified in two groups according to their dd-cfDNA RCV (>73%) or to their dd-cfDNA levels at the time of ALAD (acute lung allograft dysfunction) (>1%). The test performances are better with the RCV, with a sensitivity of 87% vs. 50% and the same specificity at 78%. The RCV seems to be a more reliable indicator of ALAD than dd-cfDNA threshold (57). Nonetheless, specificity still lacks to differentiate inflammation from infection and the need for a specific marker remains.

## 3 dd-cfDNA fragmentomic

Circulating DNA quantification can inform about allograft injury, but is not specific enough to discriminate rejection from other injuries (9, 57, 58). This is one of the obstacles still hampering the routine use of cfDNA, and specific criterions are required.

The size profile of the circulating cell-free DNA may be a precious source of information. The fragmentomic profile of cfDNA in blood can discriminate rejection from other injuries, but also different types of rejection. Agbor-Enoh et al. reported smaller dd-cfDNA fragments (<120 bp) in AMR background compared to ACR and controls (41). Moreover, one month after transplantation, the percentage of small fragments (%80–120 bp) is correlated to infection and can discriminate infected vs. non-infected patients. With a threshold of small dd-cfDNA fragments of 3.7%, 12/14 infection patients are correctly identified (85%), with PPV and NPV of 61.1% and 94.6% respectively. Moreover, the combination of dd-cfDNA levels (>1.72%) and the percentage of 80–120 bp fragments (>3.7%) for infection detection yields PPV and NPV of 100% and 82% respectively. As reported earlier, %dd-cfDNA alone cannot discriminate infection from rejection but adding the percentage of small fragments allows to differentiate infection or rejection lung injury. With a PPV of 100%, this suggests that patients with >3.7% of small fragments dd-cfDNA are infected, while AR is more frequent when <3.7% of small fragments (9).

Quantification of donor-derived cell-free DNA represents a great step towards the non-invasive monitoring of allograft rejection, but may not be sufficient in terms of specificity, especially in the context of concomitant infection. Further studies are required to determine the dd-cfDNA features relevant in SOT, to establish the correct diagnosis. Over the past few years, the rise of interest in epigenetics also showed a potential use in organ transplantation by increasing the specificity of predictive models.

## 4 Epigenetic contribution in SOT

Epigenetics refers to changes in gene expression without any modification of the genome sequence. Through different levels of regulation, epigenetics plays a key role in various physiological and non-physiological states and are dynamically adapted depending on environmental signals. Growing evidence shows that epigenetic regulation of several immunity-related genes occurs after solid organ transplantation, some of them being correlated to the fate of the transplant overall. Transplantation is very conducive to epigenetic regulation, and the analysis of cfDNA obtained from liquid biopsies can reflect these epigenetic modifications and thus be informative of the dynamic evolution of the transplant acceptance, allowing a real time and dynamic estimation of the patient's status.

### 4.1 Mechanisms of epigenetic regulation

Epigenetic marks regulate the level of chromatin compaction and thus condition gene expression. The most studied are DNA methylation and histone post-translational modifications. These modifications are reversible and dynamic and are summarized under the concept of the epigenome. Most epigenetic regulations result in the opening or closing of the chromatin fiber.

#### 4.1.1 Epigenetic regulation at the DNA level

Among epigenetic modifications, the most stable is DNA methylation. This is currently the most extensively studied epigenetic feature. It consists in the transfer of a methyl group from S-adenosylmethionine (SAM) to the fifth carbon of a cytosine ring engaged in a CG dinucleotide to obtain a 5′-methylcytosine (5-mC). This chemical reaction is catalyzed by three DNA-methyltransferases (DNMT): DNMT3a and DNMT3b involved in *de novo* methylation of the DNA strand, and DNMT1 responsible for the conservation of existing methylation, mainly during the replication process (59). Of note, although DNA methylation is very stable, it can be reversed actively by the action of specific enzymes, or passively through a “dilution” across DNA replication cycles (59, 60).

DNA methylation mainly occurs in CpG dinucleotide (61) dispersed throughout the genome while CpG-dense regions of 0.2–2 kb called CpG islands, composed of 60%–90% 5-mC usually remain free of methylation (62). CpG islands are located less than 1 kb afar from transcription start sites or encompass these sites and contribute to the transcriptional state of genes (2, 59). The abundance of methylated cytosines in gene promoters is correlated to gene repression (63).

**Figure 1:**
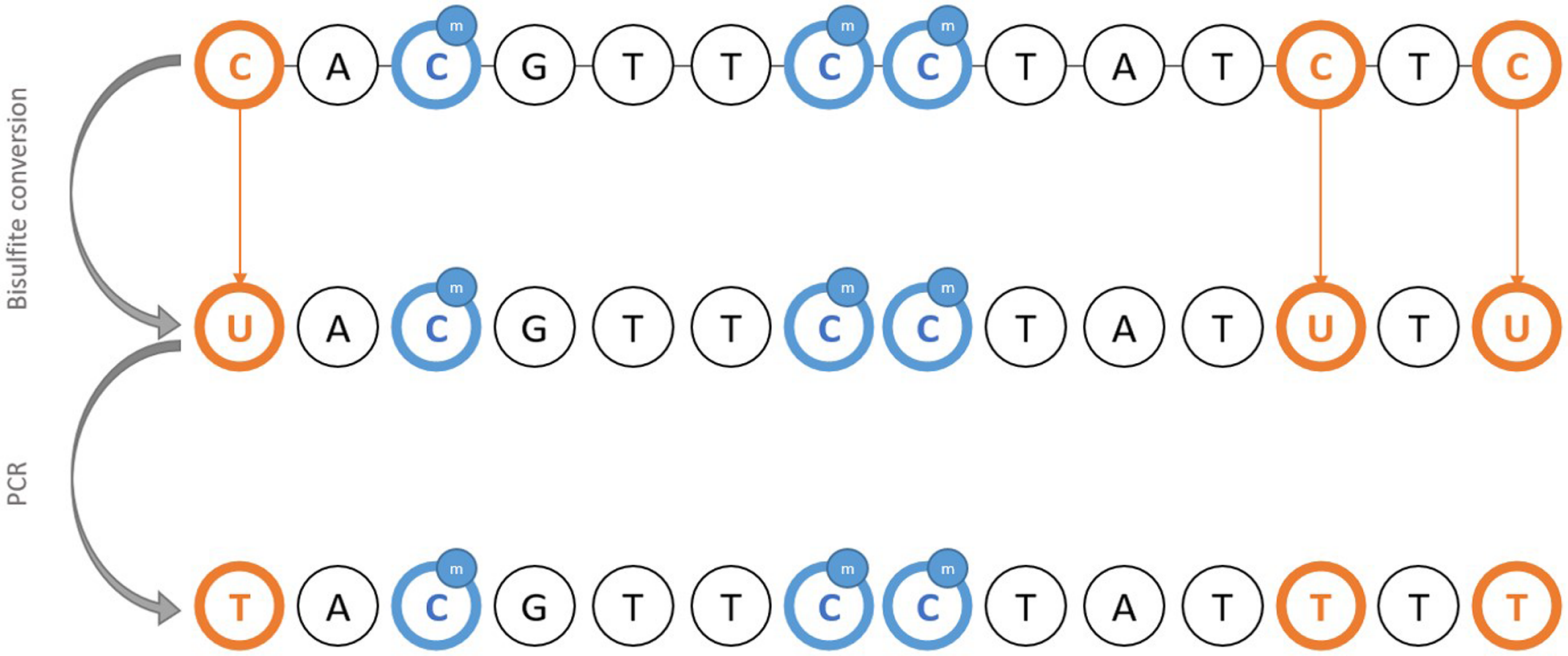
Schematic representation of the principle of bisulfite DNA conversion for the methylation study.

Classical PCR methods fail to detect methylation since base pairing is identical regardless of the methylation status of cytosine. Thus, DNA requires pre-treatment using sodium bisulfite, considered a gold standard for the detection of methylation. Basically, unmethylated cytosines are converted to uraciles through oxidative deamination, leaving methylated cytosine intact (64, 65). Therefore, it is possible to infer the initial methylation status from sequencing products after PCR amplification of bisulfite-treated DNA (Figure 1). The downside of using bisulfite pre-treatment is the DNA degradation, which prevents it from being reused for further applications. An alternative is to use methylation-sensitive restriction enzymes (66), and analyzing the digestion products to infer the methylation status where cleavage occurred.

Bisulfite-treated DNA can then be amplified through methylation-specific PCR (MSP), able to detect rarely methylated sites against a strong background of unmethylated cytosines, sometimes referred as MethyLight PCR. Based on fluorescence, MSP allows detection and quantification of methylation by using, after a bisulfite conversion of DNA, methylation-specific primers and probes designed to specifically amplify methylated or unmethylated-DNA (67, 68). To further increase the method's sensitivity, MethyLight ddPCR was adapted on digital PCR technique to be used with biological fluids containing low DNA concentrations. The quantification limit was reported to be 25-fold inferior to classical MethyLight PCR, and 20-fold inferior quantification limit (69), making this technique suitable to study cfDNA in liquid biopsies.

#### 4.1.2 Epigenetic regulation at histone level

Histones are globular basic proteins of around 200 amino-acids, rich in Arginine (R) and Lysine (K) (70), associated in octamers to form the nucleosome core (dimers of H2a, H2b, H3 and H4), around which DNA wraps. Free NH_2_ tails are vulnerable to chemical modifications (70–73) that will disrupt DNA-histone bonds, thus modifying chromatin conformation.

**Figure 2:**
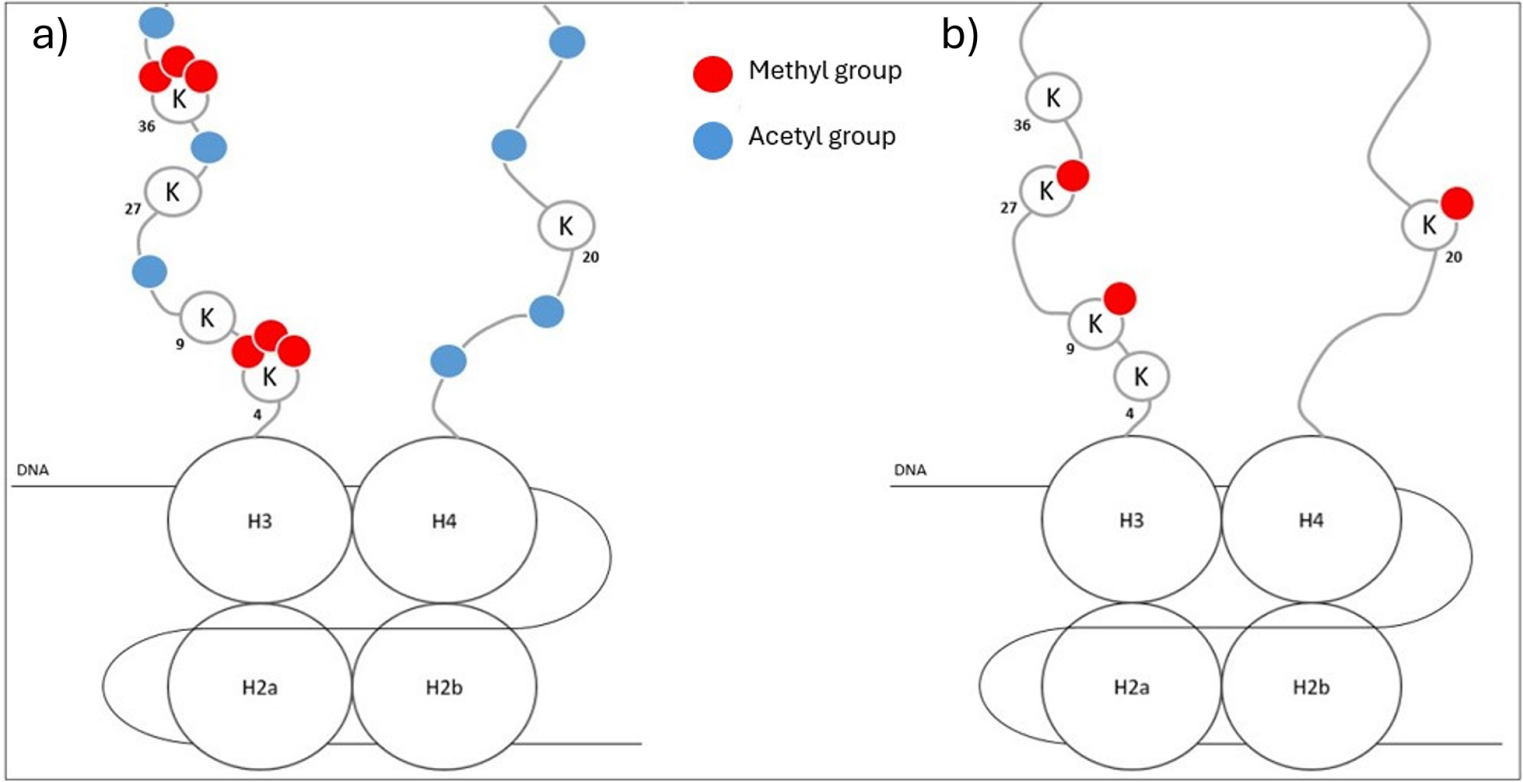
Global representation of characteristics of transcriptionally active **(a)** or inactive **(b)** chromatin.

Several major histone modifications are identified with a clear consequence on the chromatin structure and gene expression. Transcriptionally active chromatin is mostly characterized by high NH_2_-term acetylation, tri-methylation of lysine 4 on H3 (H3K4me3) (71), and acetylation of H3K27 (H3K27ac) (74). Gene bodies of transcriptionally active genes are associated with trimethylated H3K36 (H3K36me3) (74). On the other hand, transcriptionally inactive chromatin is marked with global histone hypoacetylation, and methylation of H3K9, H3K27, and H4K20 (71, 73, 74) (Figure 2). Another way histones can affect epigenetic regulation is through nucleosome shifting. Nucleosomes can slide along DNA (*cis*) or transfer the histone core to another DNA strand (*trans*) [Doyen, 2006 (75)], modifying gene accessibility and thus gene transcription and expression (2).

The study of histone modification relies on classical protein analysis techniques. In 2007, Shechter et al. presented several techniques and standard protocols for the analysis of histone and chromatin modifications (76), including very detailed protocols for acid extraction of histones, reversed-phase HPLC (RP-HPLC), and western blotting.

The enrichment analysis of specific loci requires ChIP (chromatin immunoprecipitation) with a site-specific antibody, targeting the chemical modification of interest (74, 77). Co-immunoprecipitated DNA can be submitted to qPCR, microarray analysis, or deep sequencing.

### 4.2 Involvement of epigenetics of cfDNA in solid organ transplantation

In the context of solid organ transplantation (SOT), epigenetics has two interesting features: the identification of the tissue of origin of cfDNA based on the epigenetic signature of DNA, and the assessment of the clinical status of the recipient. Epigenetic dynamics regulate the involvement of the immune system and play a key role in the success of transplantation (Table 3).

**Table 3:** Summary of the main reported epigenetic marks in different injury contexts in transplantation.

Context	Hypo/demethylated genes	Hypermethylated genes	Histone marks	Source
Inflammation	IL-6, AIM2, USP2, TMEM49, SMAD3	SOCS-1, EEF2, MGMT		Gonzalez-Jaramillo et al. (78)
IRI	C3 promoter	YBX2, Foxp3		Singer et al. (79)
Acute rejection	IL-17, RORC	IL-2, mTOR pathway genes	H3K4me3, H3K27me3	Vasco et al. (72) Zhang et al. (80) Zhu et al. (81) Suárez-Álvarez et al. (82)
Immune tolerance	HLA-G, Foxp3,		Hyperacetylation of Treg specific methylated region, H3K9ac,	Singer et al. (79) Moreau et al. (83) Bestard et al. (84)

#### 4.2.1 Tissue of origin footprint

Several studies focused on the identification of the source tissue of cfDNA relying on tissue-specific methylation patterns (85). Using genome-wide bisulfite sequencing of plasma DNA, several researchers provided “tissue maps” of plasma DNA and built methylome atlas with specific methylation profiles for each cell type or tissue (86, 87) allowing to deduce the contribution of each of them in the plasma pool of cfDNA.

In the transplantation field, this can be useful to assess the proportion of cfDNA derived from the donor organ. In a study on liver transplant recipients, the authors found a strong correlation between the dd-cfDNA fraction deduced by methylation tissue mapping determined on the basis of SNP (87).

**Figure 3:**
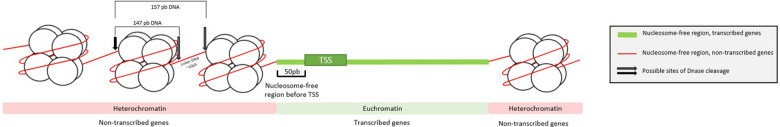
Representation of nucleosome occupancy regarding the expression or repression of genes. TSS, transcription starting site.

As actively transcribed genes are depleted of nucleosome about 50 bp upstream from their transcription start site (2, 59), nucleosome positioning varies between cell types (2), leading to a precise pattern of expressed genes (2, 88) (Figure 3). This has been used to demonstrate the main hematopoietic origin of cfDNA (88).

Since DNA is protected from cleavage when associated with proteins, some hypothesized that cfDNA carries the nucleosome footprint of the tissue it originates from, of which the fragmentomic profile is representative. To test this hypothesis, Snyder et al. performed a series of tests based on deep cfDNA sequencing and fragment endpoint alignment. They confirmed that the endpoints of cfDNA correlated to the nucleosome positioning, with cleavage adjacent to the nucleosome, but not directly on the nucleosome core (88). In most genomic regions, nucleosome positioning is tissue-specific mirroring gene expression profile (2), and DNase cleavage sites can inform on nucleosome position distribution (1).

Recently in 2022, Zhou et al. also relied on the fragmentomic profile of cfDNA to infer the methylation status of each CG dinucleotide and thus the epigenetic regulation at a single base level. After bisulfite DNA treatment, they found that densely methylated CpG tend to be enriched in 5′CGN ends (N being any nucleotide) while unmethylated CpG sites are enriched with 5′NCG end motifs. Thus, a ratio of CGN/NCG fragment end-motifs is representative of methylation-specific cleavage. Finally, they assessed the relevance of using the CGN/NCG ratio to deduce the tissue of origin of cfDNA on a murine hepatic transplantation model and were able to discriminate the dd-cfDNA proportion according to the CGN/NCG ratio. Thus, the fragmentomic profile of cfDNA can be informative of the tissue of origin depending on specific hyper or hypo-methylated sites of the tissue of interest (89).

#### 4.2.2 Ischemia-reperfusion injury (IR)

As epigenetic regulation is dynamic and vulnerable to changes in the environment, cfDNA carries epigenetic hallmarks reflecting the immune system activity in recipient after a transplantation and may allow to anticipate the recipient's response to the allograft. Studies identified relevant epigenomic patterns involved in the immune system activation or regulation in patients at different key events following transplantation. The aim is to identify recurrent patterns, specific to rejection or acceptance, based on epigenetic behavior of specific genes.

Ischemia-reperfusion injury (IRI) is caused by the allograft's cells hypoxia before the transplantation, which accumulate metabolic wastes causing inflammation and fibrosis (90) and is a risk factor for multiple conditions that may arise later (60, 90, 91). Epigenetic regulation is thought to be a key actor of inflammation-related pathways (78). DNA methylation plays a key role in IRI, and several differentially methylated sites are identified related to pro-inflammatory genes and molecular pathways. More generally in inflammation, Gonzales-Jaramillo et al. reviewed twenty-four studies identifying differentially regulated markers. Briefly, inflammation is associated with a global hypomethylation of DNA, and hypomethylation of several pro-inflammatory cytokines genes (*IL-6*, *AIM2*, *USP2*, *TMEM49*, *SMAD3,* etc.), while other are hypermethylated (*SOCS-1*, *EEF2*, *MGMT* etc.) (78).

Back to SOT, IRI-induced aberrant demethylation on the C3 gene promoter led to tissue injury in mice kidney transplant model (92, 93). On the other hand, during ischemia reperfusion induced acute kidney injury, hypermethylation of *YBX2* promotes transition to chronic-kidney disease (CKD). Demethylation of *YBX2* induced with the methyltransferase inhibitor 5-azacitidine, prevented the transition to CKD while also attenuating fibrogenesis (91).

In lung transplantation, few data are available. Very recently in 2023, Liu et al. demonstrated that DNA methyltransferases (DNMT) inhibitors attenuated lung injury and inflammation, while DNA demethylation enzyme inhibition worsened lung injury, confirming the implication of DNA methylation in IRI (94). DNMT inhibitors also seems to accelerate lung inflammation resolution through demethylation of the *Foxp3* locus in regulatory T cells (Tregs) (79).

The key mechanism of IRI is the inflammation induced by cytokine production and leucocytes recruiting leading to the activation of apoptosis of injured cells (95). Several differentially methylated regions (DMR) have been identified. Few data are available about other epigenetic mechanisms like histone modification. Globally, the analysis of DNA methylation at specific locus of inflammatory genes may be relevant to detect the setting of inflammation.

#### 4.2.3 Acute rejection

The next critical step after SOT is the potential development of acute rejection (AR), whether being humoral (AMR) or cellular (ACR). Epigenetic programming can regulate the differentiation and activation of immune cells like dendritic, T and B cells (72) and lead to the initiation of acute rejection. Several pathways are identified to be under epigenetic regulation with DMR specific to the clinical status of the recipient.

DNA methylation regulates IL2-mediated T cells activation, identified as a leukocyte growth factor. IL-2 promoter is hypermethylated and thus inactive in naïve T cells, while hypomethylated and strongly acetylated in active T cells (72). In rats, H3K4 trimethylation (H3K4me3) is shown to increase in peripheral blood mononuclear cells in the context of AR (80), and the use of histone methyltransferase inhibitors suppresses the alloimmune reactivity of T-cells, improving AR in kidney-transplanted rats (96).

The mammalian target of rapamycin (mTOR) pathway is a central signaling pathway controlling the epigenetic rewiring of myeloid cells (97), as well as proliferation, growth, and cell survival (72, 81). Several genes related to the mTOR pathway are hypermethylated in AR-induced allograft dysfunction in renal transplant recipients (81). Moreover, pharmacological inhibition of mTOR specifically blunt pro-inflammatory cytokine expression (IL-6 and TNF-α) *in vitro* (97), and demethylation of down regulators of mTOR with DNA methyltransferases also improved the inflammatory injury on a murine model of AR (60).

Helper T-cells 17 (Th17) specifically express IL-17 and potentially contribute to allograft rejection. Th17 isolated *in vivo* are characterized by DNA demethylation of IL17 and RORC, with bivalent H3K4me3/H3K27me3 domains on the *TBX1* promoter (transcription factor), suggesting the gene is ready to be rapidly expressed under certain conditions (82).

#### 4.2.4 Immune tolerance

The goal after a SOT is to reach a state of immune tolerance, defined as a state of control or inhibition of the immune response to a foreign stimulus, leading to acceptance of the allograft without the need of immunosuppressive treatment. Epigenetics can either upregulate or downregulate the immune response to the graft and thus induce rejection or tolerance.

HLA-G is among the most studied immune tolerance actor in heart (98, 99), lung (100) and kidney (101) transplantations. This non-classical major histocompatibility complex class 1b antigen is involved in the protection of transplanted tissues through the inhibition of immune effectors (102), especially NK and T cells (103). HLA-G shows low allelic polymorphism, and can be found in soluble form (sHLA-G) (100). It can be detected in the plasma and biopsies of transplant recipients, correlated to allograft acceptance with less AR episodes and no chronic rejection (100). HLA-G transcription is enhanced by demethylating agents (83), suggesting that HLA-G promoter could be demethylated in tolerant patients. Histone methylation in a regulative region of HLA-G 450 bp upstream from start codon correlates to DNA hypermethylation in the same region, silencing HLA-G expression (103). Moreover, immunosuppressive drugs would influence HLA-G expression, with sHLA-G increasing in patients treated with Everolimus and Tacrolimus in cardiac and renal transplantations respectively (104, 105). Finally, patients treated with belatacept, a recombinant molecule used to prevent AR after kidney transplantation, show an increase in sHLA-G levels compared to patients treated by calcineurin inhibitors or healthy donors.

Different cell types are engaged in the regulation of the immune response in SOT, and thus in immune tolerance of the allograft. Regulator T cells (Treg) are essential in immune homeostasis due to their role in peripheral tolerance (106). These cells are characterized by the expression of the *Foxp3* (Forkhead Box Protein 3) transcription factor. Several studies showed that *Foxp3* demethylation is associated with a higher expression of Tregs intra-graft, and a favorable long-term outcome for the allograft (79, 84), as well as histone hyperacetylation (82) in the Treg-specific demethylated region (TSDR).

An interesting mechanism in allograft acceptance is the exhaustion of T cells, a functional silencing leading to exhausted T cells (Texh). This occurs in response to a prolonged exposition to an antigen, limiting T cells ability to release cytokines and resulting ultimately to impaired ability (107). Higher levels of Texh are associated to a better renal function (107). Texh epigenetic program is robust and stable, distinct from effector and memory T-cells (108). When exposed to persistent levels of antigens, T cells upregulate PD1 and a repressing methylation prevent exhausted T cells to respond to immune checkpoint blockage (109).

Dendritic cells (DCs) are essential for innate and adaptive immune response but DCs can induce immune tolerance in the absence of signal (82). During monocyte derived tolerogenic DCs differentiation, epigenetic mechanisms are involved, and guide the becoming of DCs as activated or tolerogenic. Monocyte differentiation in DCs is regulated by acetylation of H3K9 and the loss of repressive marks H3K9me3 and H4K20me3, along with DNA methylation (110).

## 5 Conclusion

The clinical use of cfDNA is still subject to various obstacles, which must be overcome before a reliable and robust diagnostic test can be developed. First, methods standardization for both sampling and analyzing cfDNA are mandatory to allow reproducibility of tests, and thus improve the quantity and quality of data relative to the diagnostic relevance of cfDNA. Moreover, the various methods used yield different sensitivity and specificity, implying the need to improve the algorithms of classification, especially by increasing cohort sizes, partly made possible by method standardization to share results. Finally, the correlation between circulating DNA and the veracity of clinical rejection is not always easy to establish, as evidenced by the threshold values of %ddcfDNA which, even if close, are never identical from one study to another, and can be impacted by confounding factors such as infection.

Epigenetics holds promises and can bring solutions to greatly increase sensitivity, specificity and reliability of diagnosis with cfDNA. It may allow discrimination between rejection and infection or other confounding factors and avoids non-standardized extraction and quantification methods. Efforts remain to be made towards identifying specific epigenome patterns characteristic of acute rejection being cellular or humoral, infection, and other conditions, allowing early and reliable identification of rejection mechanisms activation.
